# An Unusual Case of Prostate Carcinoma With Metastasis to the Iliopsoas Muscle and Nerve Root Impingement

**DOI:** 10.7759/cureus.16286

**Published:** 2021-07-09

**Authors:** Joyce Kong, Vikram Sumbly, Zarwa Idrees, Khalid Mahmood

**Affiliations:** 1 Internal Medicine, Icahn School of Medicine at Mount Sinai / New York City (NYC) Health + Hospitals / Queens, Jamaica, USA

**Keywords:** prostate carcinoma, metastasis, iliopsoas muscle, skeletal muscle, association: prostate cancer

## Abstract

The majority of prostate cancer cases carry a favorable prognosis due to various screening protocols and the progression of surgical/medical techniques. Prostate cancers that metastasize to the skeletal system bear worse five-year survival rates as they are indicative of widespread dissemination. There are very few cases of prostate cancer invading the iliopsoas muscle described in the medical literature. Here, we present the case of a 61-year-old male who was diagnosed with prostate cancer with metastasis to the bones and iliopsoas muscle. Given the advanced presentation of his disease, the patient underwent a prostate biopsy. He was initiated on bicalutamide and transitioned to leuprolide and docetaxel with eventual radiation therapy.

## Introduction

Prostate cancer is the most commonly diagnosed neoplasm among men in the United States [1]. Although most cases of prostate cancer have a favorable outcome, they still represent a significant portion of cancer deaths worldwide. Indeed, it is estimated that approximately 5.6% of all cancer deaths in 2020 were related to prostate cancer [2]. The wide array of symptoms experienced by prostate cancer patients will depend on the following factors: the size and location of the tumor and affected organ [3]. If left untreated, prostate cancers can metastasize to the bones or lymphatic system [3]. Atypical sites include the liver, lungs, and brain [3]. Skeletal metastasis is a rare phenomenon with the majority of tumors originating from the pulmonary, gastrointestinal, and urological systems [4]. Prostate cancer metastasizing to the iliopsoas muscle is a very unusual occurrence that has only been documented in scant case reports. Here we present the case of a male who was newly diagnosed with prostate cancer with metastasis to the axial skeleton and iliopsoas muscle.

## Case presentation

A 61-year-old Haitian male with a past medical history of hypertension presented to the emergency department (ED) with excruciating intermittent left-sided paraspinal lumbar pain with numbness and tingling radiating to the left lateral knee for four days. The pain was described as a 10 out of 10 electric shock sensation. He denied trauma to the area, family history of cancer, personal history of prostate disease, any prior colonoscopy, or history of steroid use. He admitted to increased urinary frequency but denied straining, dribbling, or hesitation. He denied smoking or recreational drugs but endorsed drinking alcohol socially. He also denied any home medications. His vitals showed a blood pressure of 168/98 mmHg, a heart rate of 121 beats/min, a respiratory rate of 18 breaths/min, and oxygen saturation of 96% on room air. The electrocardiogram (EKG) showed sinus tachycardia with a heart rate of 121 beats/min. On initial assessment, the patient had mild tenderness to palpation of the left lumbar paraspinal muscle and the lateral thigh region.

**Figure 1:**
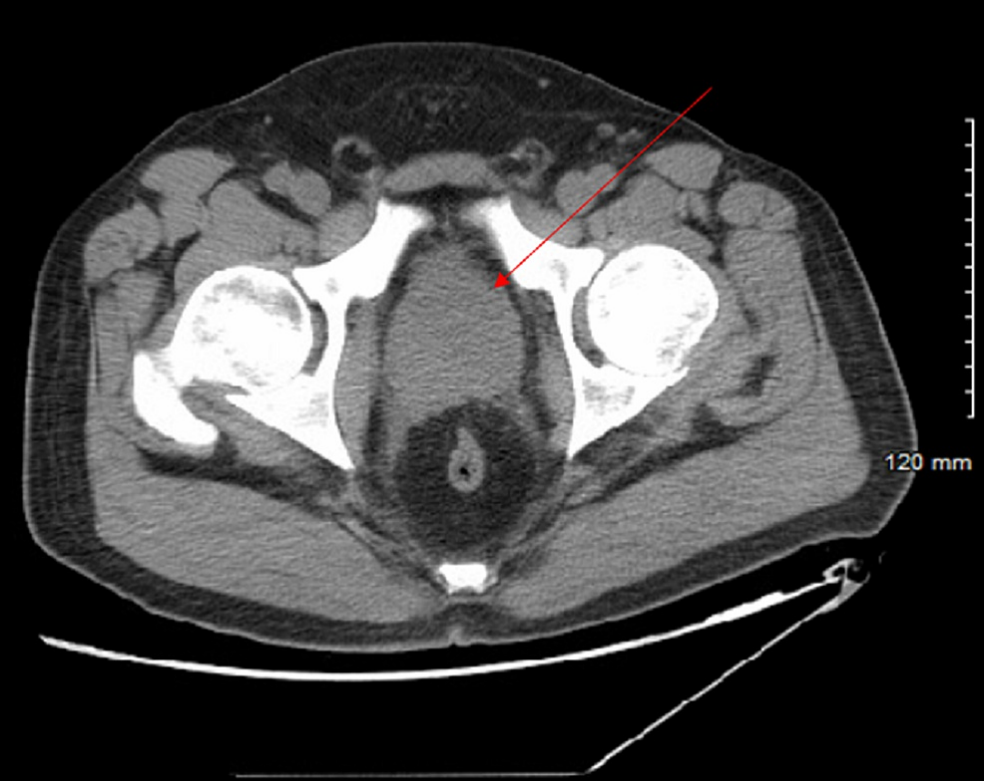
Enlarged prostate measuring 5.6 cm transverse by 5.5 cm AP. AP, anteroposterior

**Figure 2:**
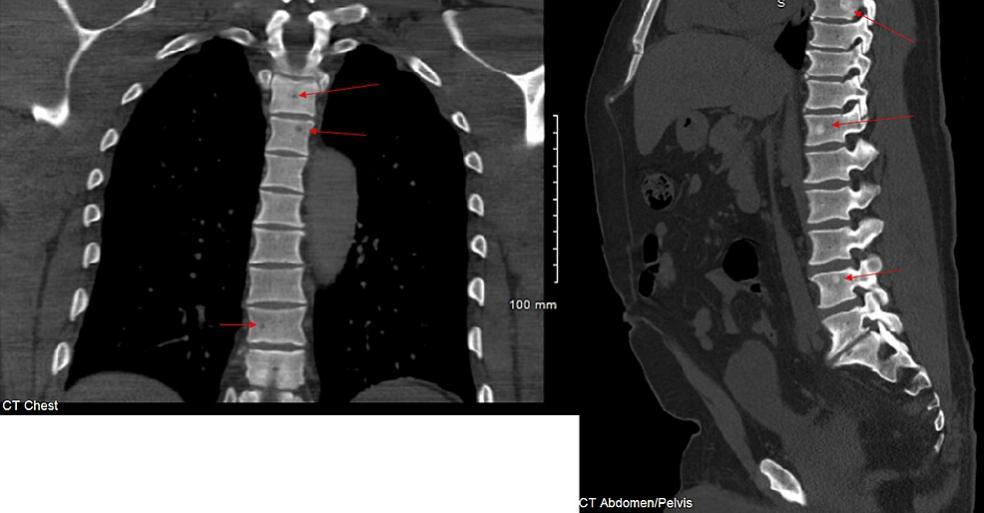
CT scan (bone window): bone lesions likely secondary to metastatic prostate cancer.

**Figure 3:**
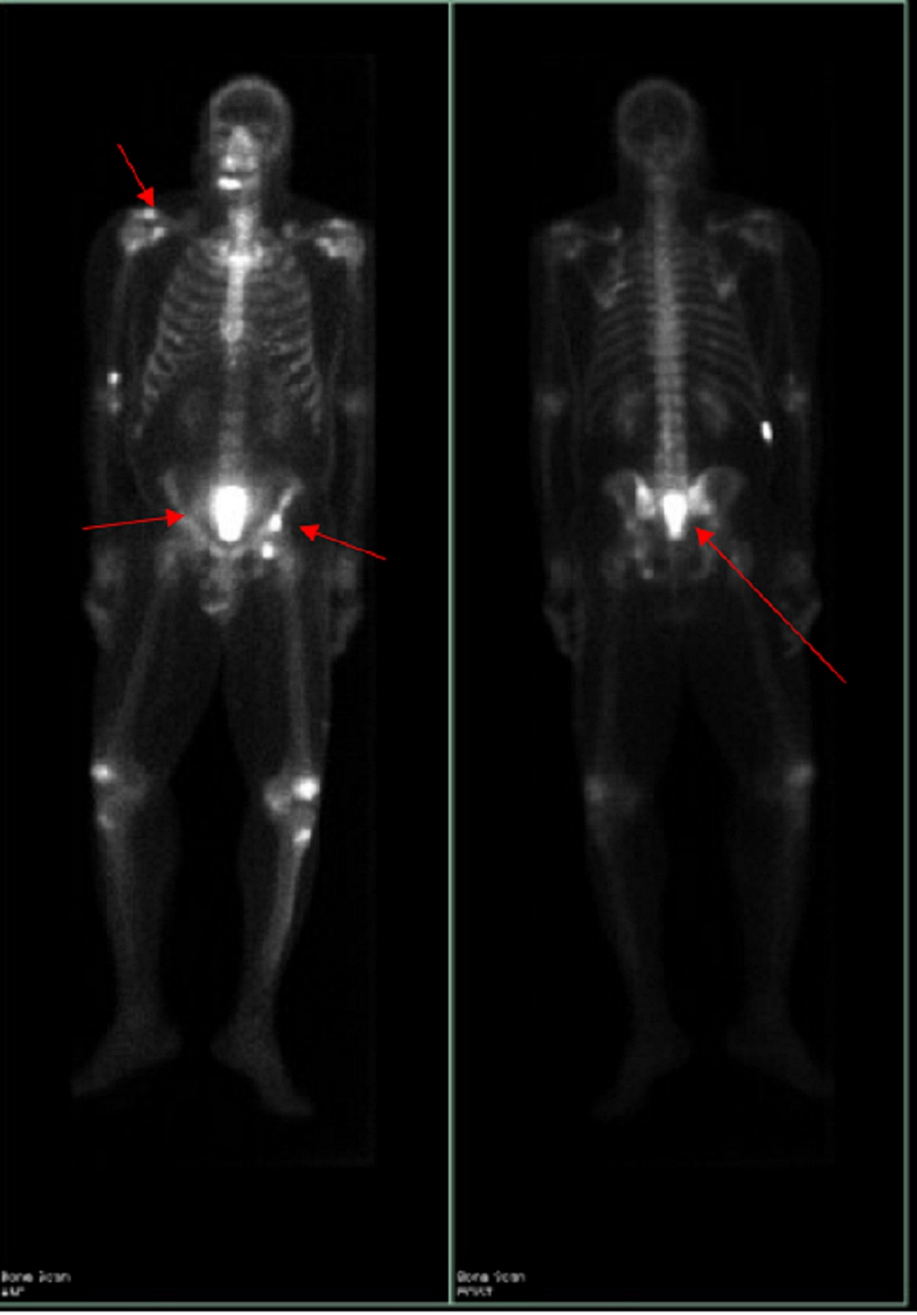
PET scan showing metastasis to bone. PET, positron emission tomography

**Figure 4:**
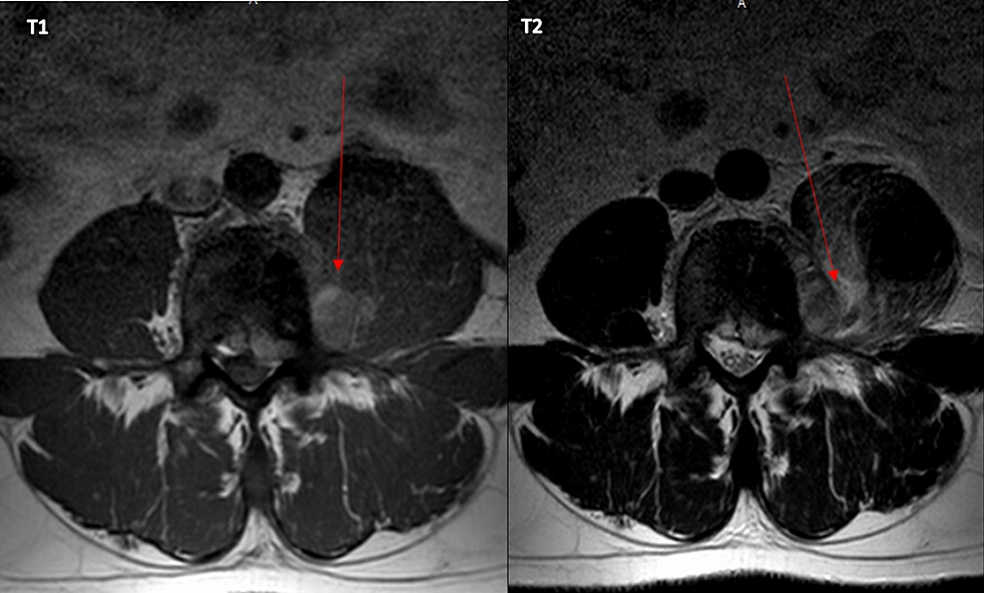
Metastatic lesion within the left psoas muscle around the L4 region associated with an epidural lesion.

Initial laboratory findings showed normal renal function and grossly normal complete blood count. Urinalysis was negative. Other labs revealed a procalcitonin of 0.06 ng/mL (within normal limit), ferritin of 378 ng/mL (within normal limit), and a C-reactive protein (CRP) of 1.0 mg/L (within normal limit). A1C was 7.7. The hepatic function panel showed elevated alkaline phosphatase 183 U/L (reference range 40-129 U/L). Prostate specific antigen (PSA) total was 614 ng/mL (reference range 0-4 ng/mL). Serum and urine protein electrophoresis were negative. CT scan showed an enlarged prostate and a spine with numerous sclerotic lesions (Figures 1-2). Similar osseous lesions were also visible on the positron emission tomography (PET) scan (Figure 3). However, MRI of the lumbar spine showed a large epidural enhancing lesion along the left side extending into the anterior/anterolateral aspect of the spinal canal as well as along the medial aspect of the left psoas muscle with significant surrounding edematous changes along the length of the left psoas muscle as well and associated swelling (Figure 4). After being consulted, neurology recommended the patient to be started on lacosamide 100 mg every 12 h and dexamethasone taper for better pain control. The patient underwent a transrectal ultrasound prostate biopsy and was started on bicalutamide 50 mg oral daily per urology recommendations given the high likelihood of advanced malignancy. Prostate biopsy obtained at 12 sites showed 11 of 12 sites as prostatic adenocarcinoma with a combined Gleason score of nine. 

**Figure 5:**
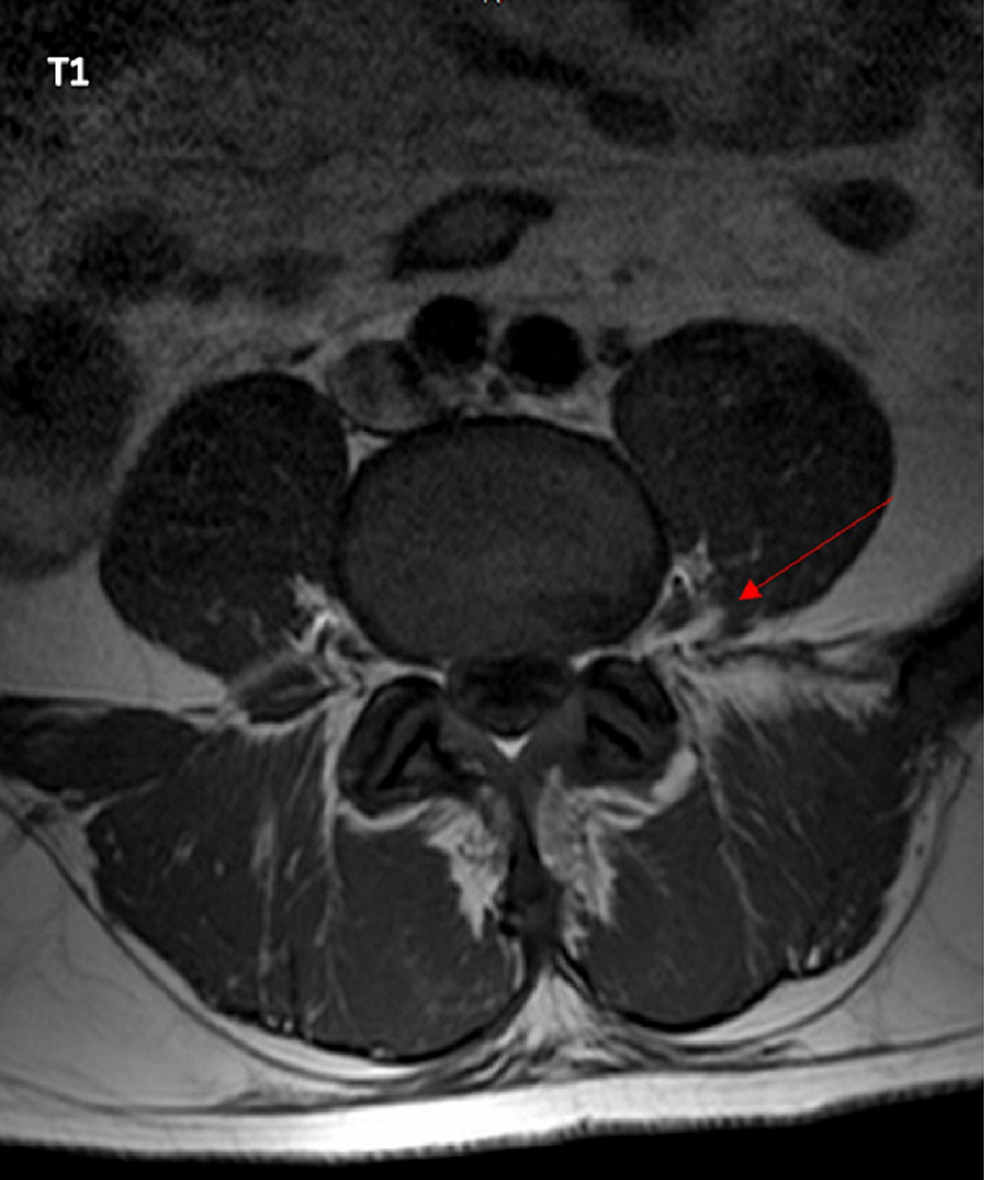
Metastatic lesion along the medial aspect of left psoas muscle near the L4 nerve root as well as the anterior margin of the thecal sac and neural foramen.

After being discharged from the hospital, the patient followed with urology and medical oncology who started him on a leuprolide 45 mg six-month depot subcutaneous regimen and docetaxel 75 mg/m2 monthly regimen, respectively. The PSA value was monitored every month with an appropriate response; the most recent PSA was within the normal range. In March 2021, he completed six cycles of docetaxel and pegfilgrastim. Repeat MRI showed psoas muscle involvement was improved but there is still abnormal enhancement along the medial aspect of the left psoas muscle at the L4 level along the left side with additional enhancement also seen posterolaterally and surrounding adjacent L4 nerve root as well as the anterior margin of the thecal sac and neural foramen (Figure 5). Radiation oncology recommended palliative radiation therapy at the L3-S1 region given new MRI findings. In April 2021, he completed a course of 30 Gy in 10 fractions (fx) to his back. He is currently following oncology, radiation oncology, and urology closely. 

## Discussion

Prostate cancer remains one of the most commonly diagnosed neoplasms in men. According to GLOBOCAN 2020, prostate cancer represented 7.3% of all newly diagnosed cancers worldwide and 14.1% of all newly diagnosed cancers in men. Approximately 7% of all cancer-related deaths are linked to this specific type of tumor [5]. Tumorigenesis involves mutations in several genes such as TP53, BRCA1, BRCA2, and HOXB13 [6]. Early-stage tumors are often confined within the prostate gland and cause urinary retention/hesitancy, nocturia, hematuria, impotence, and weight loss [7]. Once metastasis occurs, the symptoms experienced by patients will mainly depend on the final location of cancer. 

Prostate cancer metastasis is thought to be triggered by metastasis-inducing cells (MIC) which have the ability to recruit and reprogram dormant and/or bystander cells (e.g. bone marrow stromal cells, endothelial cells, immune cells, and fibroblasts) within the tumor microenvironment [8]. This process has been shown to disrupt the c-Myc/Max and AP4 biochemical pathways via RANK-mediated signaling [8]. Most prostate cancers grow within the peripheral zone of the prostate gland before metastasizing to the bone or lymph nodes. Only in very rare circumstances will prostate cancers metastasize to the muscular system. Studies have shown that myocytes are resistant to neoplastic invasion due to their innate ability to produce a low molecular weight factor and adenosine [9]. Fibroblasts have also been observed to protect striated myocytes by engulfing tumor cells due to their ability to sense “foreign” bodies [10].

Metastatic lesions to the iliopsoas muscle can be easily missed if not adequately looked for. Most patients complain of pain, which can erroneously be dismissed as “regular” muscular pain; that is why advanced imaging is a must. MRI and PET scans are excellent tools in determining if widespread metastasis has occurred due to their high sensitivity and specificity [11-12]. In our patient, an MRI was sufficient to confirm a final diagnosis of metastatic prostate cancer with impingement of the spinal nerve around the L4 region. The PET scan further solidified this diagnosis as there were osteoblastic lesions seen throughout the patient’s axial skeleton.

To the best of our knowledge, there have been very few cases of prostate cancer with iliopsoas muscle metastasis and nerve impingement [13-14]. According to Kelly et al., the current annual incidence of metastatic prostate cancer in the United States is slightly less than 19/100,000 person years [15]. The associated five-year survival is approximately 28% [16]. Advanced prostate cancer can be treated by hormone therapy, immunotherapy, or chemotherapy. Patients are often started on gonadotropin-releasing hormone (GnRH) analogs such as leuprolide, triptorelin, or goserelin which suppress the testicular production of testosterone [17]. These antiandrogens can also be given in combination with bicalutamide which inhibits the systemic production of testosterone [17]. Patients who suffer from hormone therapy-resistant prostate cancer can be given docetaxel, a chemotherapeutic agent, which has been shown to prolong the lifespan of men no longer responding to antiandrogen therapy [17]. Cabazitaxel is a second-line chemotherapy that can be given to patients with progressive disease despite being on docetaxel [17]. Sipuleucel-T and pembrolizumab are immunotherapeutic drugs that have also been shown to increase the lifespan of patients with terminal prostate cancer [18-19]. 

## Conclusions

The vast majority of prostate cancer cases consist of tumors, which are considered to be “slow-growing” and “slow-progressing.” These can be properly managed via active surveillance. This case serves as a reminder about the importance of prostate cancer screening as dissemination to musculoskeletal sites bears a terrible prognosis. It is important to keep this in mind whenever a male patient presents with urinary disturbances accompanied by abnormal muscle pain. Earlier detection in our patient might have prevented metastasis and increased his chances of survival.
